# The Wishes, Perceived Barriers, and Support Needs of People Living in Persistent Poverty

**DOI:** 10.1177/10497323241309803

**Published:** 2025-03-28

**Authors:** Lincy Scholten, Sandra H. H. Schel, Linda van den Dries, Gerdine A. J. Fransen-Kuppens, Renée de Vet, Judith R. L. M. Wolf

**Affiliations:** 1 Department of Primary and Community Care, Research Institute for Medical Innovation, Radboud University Nijmegen Medical Center, Nijmegen, The Netherlands; 2 Research centre for Social Innovation, 8119 Utrecht University of Applied Sciences, Utrecht, The Netherlands

**Keywords:** poverty, wishes, barriers, support needs, socio-economic security

## Abstract

This study explores the wishes, perceived barriers, and support needs of people living in persistent poverty. These remain undetermined, yet are essential for developing effective support for this group. We conducted semi-structured video call interviews with people living in persistent poverty (*n* = 14), peer workers (*n* = 5), and practitioners (*n* = 5) in five municipalities in the Netherlands. The findings show that wishes revolve around improving individuals’ socio-economic security, their (loved-ones’) well-being, and relatedness. The realization of these wishes is hindered by socio-economic barriers, support structures, psychological processes, and an intergroup reality gap. Participants therefore advocated for structural changes in policy and practice. These include more income, a focus on poverty prevention, easily accessible and tailor-made support, and bridging the gap between the living and system world. In addition, service provision for people living in persistent poverty should prioritize the quality of the working alliance, self-direction, a holistic approach, and the engagement of peer workers.

## Introduction

In 2022, approximately 72.7 million people in the EU were living in poverty (at-risk-of-poverty rate, Eurostat, 2024). In the Netherlands, the number of individuals living in poverty is estimated at 2.5 million, which is about 1 in 7 people (at-risk-of-poverty rate, Eurostat, 2024). About 40% of the Dutch living in poverty had been living in poverty for at least four consecutive years (low-income-threshold, Statistics Netherlands, 2023). Poverty has detrimental consequences for the quality of one’s daily life. It is related to worse physical and mental health outcomes, especially when persistent (Kahn & Pearlin, 2006; Richardson et al., 2013), to higher stress-levels (Haushofer & Fehr, 2014), and to lower levels of self-regulation and perceived control (Evans & Kim, 2012; Price et al., 2002). Poverty has also been associated with impaired cognitive functioning (Mani et al., 2013), weaker social relationships (Mood & Jonsson, 2016), feelings of social exclusion (Marttila et al., 2010), and domestic violence (Marttila et al., 2013; Taylor & Barusch, 2004). These disadvantages tend to accumulate (Marttila et al., 2013; Taylor & Barusch, 2004) and shape the opportunities of future generations, as growing up in poverty predicts a lower well-being in adulthood (Crouch et al., 2020). Poverty is consequently considered both a widespread and enduring public health problem (Price et al., 2018) and an extreme form of social exclusion (Watson et al., 2016). It is therefore crucial to facilitate appropriate and effective support to improve the quality of life of people living in persistent poverty.

Over the years, valuable studies have explored what appropriate and effective support for people living in persistent poverty entails by assessing the impact of interventions on their well-being (e.g., experiments with more autonomy and trust-based social assistance, cash transfer experiments, and service provision interventions such as Mobility Mentoring®; Betkó, 2023; McGuire et al., 2022; Thomson et al., 2022; Washington State, Department of Children, Youth, and Families, 2018). With this study, however, we aim to take a step back and first explore what it is that people living in persistent poverty wish to see differently in their lives, irrespective of such interventions. Founding support on their wishes is imperative because the ability and opportunity to pursue one’s own wishes has been associated with better well-being and performance (Deci & Ryan, 2000; Sheldon et al., 2004; Vansteenkiste et al., 2004). In addition, wishes function as key motivators in the recovery process (Lai et al., 2021; Wolf & Jonker, 2020).

The wishes of people living in persistent poverty, however, remain largely unknown. Wishes refer to individuals’ needs (i.e., that what is essential for survival) and their wants (i.e., individual desires). In contrast to the concept of wishes, the concept of needs has received much academic attention over the years. Maslow (1943), for instance, developed a hierarchy of needs, Doyal and Gough (1984) coined a theory of human need deeming health the most basic of needs, Deci and Ryan (2000) discerned basic psychological needs of competence, autonomy, and relatedness, and Nussbaum (2000) created a list of basic capabilities. These theories are nevertheless considered to reflect universal needs, and hence do not provide the in-depth insight into the wishes of people living in persistent poverty that is essential for developing appropriate support.

The extent to which people living in persistent poverty can realize their wishes is strongly affected by various individual and environmental barriers. Previous research has only mapped the barriers people living in persistent poverty experience for accessing healthcare and public services. These barriers include feelings of shame, fear of losing custody of their children, fear for being misunderstood and judged, perceptions that support does not match their individual needs, complex administrative procedures, and high costs (Canvin et al., 2007; Loignon et al., 2015; Williamson et al., 2006). The identified support needs of people living in persistent poverty—that is, the support they need to overcome these barriers and realize their wishes—are mostly an extension of these barriers (e.g., affordable healthcare, tailor-made support, and more flexible rules for using support) (Gupta & Blumhardt, 2016; O’Donnell et al., 2016; Williamson et al., 2006). In addition, their support needs have been addressed in evaluation studies. These studies, for instance, show that they value practitioners who are empathic and caring, who listen and respect them, and who show them that they matter (O’Donnell et al., 2016; Williamson et al., 2006). An extensive understanding of the barriers and support needs they experience in their daily lives, however, is still lacking.

In summary, to our knowledge, the wishes, perceived barriers, and support needs of people living in persistent poverty have not yet been systematically explored, nor have they been studied in relation to one another. This study aims to fill this knowledge gap by exploring (1) the wishes of people living in persistent poverty, (2) the barriers they perceive for realizing these wishes, and (3) the support they need to fulfill these wishes, from the perspectives of these individuals themselves, peer workers, and practitioners. Combining these perspectives facilitates the broad and integrated approach needed to improve support for those living in persistent poverty and to increase their quality of life (Marttila et al., 2013; Taylor & Barusch, 2004).

## Materials and Methods

### Design

This study was part of a larger research project investigating the constituents of appropriate support for people living in persistent poverty and its impact on their quality of life. The current qualitative study applied a collaborative approach by involving peer workers throughout the research process (i.e., developing information letters and data collection tools, and data collection). Semi-structured interviews were conducted to examine the perspectives of people living in persistent poverty (*n* = 14), peer workers (*n* = 5), and practitioners (*n* = 5) regarding the wishes, perceived barriers, and support needs of people living in persistent poverty.

### Ethical Approval, Quality Criteria, and Research Team

This study was exempted from formal review by the accredited Medical Review Ethics Committee region Arnhem-Nijmegen (file number 2020-7030), because it did not concern medical scientific research and involved minimal risk of harm to participants. All participants received written and verbal information about the study’s aim and procedure, and their right to withdraw at any time, without giving any reason. Participants were aware that their participation was voluntary and gave audio-recorded verbal informed consent.

At the start of the study, participants were assigned a research ID, ensuring anonymity during the process of data collection, analysis, and storage. Only the research team had access to the data and key file, which were stored separately. The research team consisted of two peer workers, five researchers, and a professor of social care (principal investigator). The interviewers were experienced in interviewing people living in vulnerable conditions, ensuring the presence of important skills required for qualitative health researchers (Morse, 2010).

### Participants and Setting

People living in persistent poverty could participate if they were at least 18 years old, had legal residence in the Netherlands, were fluent in Dutch, and were living in persistent poverty. The latter was defined as having had insufficient financial resources to pay for monthly necessary expenses for living and for social participation for at least three consecutive years between 2016 and 2020 (not-much-but-sufficient criterion, see Goderis et al., 2019). Potential participants who were unable to provide reliable information according to the recruiting practitioner (e.g., due to an active psychotic episode) were excluded from this study. Eligible peer workers had personal experience with living in persistent poverty and had received professional training in peer support work. Practitioners (either with or without personal experience with persistent poverty) were required to be professionally trained and have a paid job in service provision to people living in persistent poverty.

Participants were recruited in the five Dutch municipalities that participated in the larger research project (Groningen, Meppel, Midden-Groningen, Nieuwegein, and Nijmegen), and in surrounding municipalities.

### Recruitment

Participants were recruited via purposeful sampling. Our contacts in the five participating municipalities recruited participants directly from their network or indirectly via gatekeepers (i.e., contacts with access to the target population, such as practitioners and peer workers). They were instructed to recruit individuals that met the inclusion criteria (we relied on their professional judgement to determine whether individuals met our definition of persistent poverty) and, if possible, to select a diverse set of individuals regarding gender, age, migration and poverty background, living situation, and literacy, in order to include a large variety of perspectives. Potential participants were informed verbally about the study and received an information letter. If potential participants expressed interest to participate, their contact details were shared with the research team. A researcher then contacted them to explain the study aims and interview procedure again, to answer any questions, and schedule an interview appointment.

### Interview Guide and Data Collection

Interviews took place between March and July 2021 via video calls, as face-to-face interviews were not feasible due to restrictions following the COVID-19 pandemic. The selected communication platform (e.g., Microsoft Teams, Zoom, and WhatsApp video calls) was aligned with the preferences and skills of each participant. This flexibility ensured that technological proficiency or specific software requirements would not exclude anyone. The interviews covered the following topics: wishes, perceived barriers, and support needs. Interviews with people living in persistent poverty were conducted both by a researcher and peer worker. To explore their wishes, they were asked: “What are your wishes for the future? What do you want to change or achieve in your life?” and “why do you want to change this?”. Perceived barriers were examined by asking: “What makes it difficult for you to fulfill your wishes? What do you perceive as major bumps in the road?”. Support needs were investigated by asking: “What do you think is needed to fulfill your wishes for the future? What would help you to realize them?”. For peer workers and practitioners, the questions were adjusted to their role, for example: “What do you think are the wishes of your clients living in persistent poverty?”.

After each interview, the researcher and peer worker discussed the interview (i.e., peer debriefing) to ensure consistent performance across interviews, to check whether the interview questions should be altered, and to assess the dependability of the interview results. This did not lead to any changes in the interview guide or procedure, or to exclusion of interviews. The audio-recorded interviews took, on average, 100 minutes (range: 75–125 minutes) for people living in persistent poverty and 90 minutes (range: 74–100 minutes) for peer workers and practitioners. Interviews were transcribed verbatim and anonymized. To thank participants, people living in persistent poverty received a 50-euro gift card. Peer workers and practitioners received a 25-euro gift card.

### Data Analysis

Data analyses were supported using a qualitative data analysis software program (Atlas.ti version 7). The transcripts were analyzed using both an inductive (“conventional thematic content analysis”) and deductive (“directed content analysis”) approach, as described by Braun and Clarke (2006). More specifically, we used open, axial, and thematic coding to identify the wishes, perceived barriers, and support needs of people living in persistent poverty. In order to minimize the subjectivity of our findings, two researchers independently coded the first four transcripts line by line (two with people living in persistent poverty, one with a peer worker, and one with a practitioner). Next, they reviewed the meaning and uniqueness of the initial codes and agreed upon an initial coding scheme. This coding scheme was then discussed with a third researcher and the principal investigator, and adapted until consensus was reached. In each of the subsequent two analysis rounds, the two researchers coded five different interviews and repeated the review and adaption process described above. This resulted in a final coding scheme describing the wishes, perceived barriers, and support needs of people living in persistent poverty on three themes, which were identified during the coding process. A member check with two peer workers took place to validate the final coding scheme.

## Results

### Characteristics of Participants

The participants’ demographic characteristics are summarized in Table 1. Participants living in persistent poverty (*n* = 14) were mostly women, born in the Netherlands, and had an average age of 46 years (range: 33–63 years). Our sample included participants from different types of households and with different educational backgrounds. Some people had a paid job or volunteered. Most had been living in poverty for much longer than 3 years—even up to approximately 25 years. Self-reported causes include intergenerational poverty and life events such as job loss, illness, and divorce. These characteristics reflect the population of people living in persistent poverty in the Netherlands for gender but not for migration background, as most individuals in the population do have a migration background (Statistics Netherlands, 2023).

**Table 1.** Demographic Characteristics of Participants.

	People Living in Persistent Poverty (*n* = 14)		Peer Workers (*n* = 5)		Practitioners (*n* = 5)	
	*n*	% or Mean	*n*	% or Mean	*n*	% or Mean
Gender						
Men	4	28.6	1	20	0	0
Women	10	71.4	4	80	5	100
Age in years		46		58		40
Migration background						
No migration background	11	78.6	4	80	4	80
Migration background	3	21.4	1	20	1	20
Educational level						
Lower education	5	35.7	2	40		
Intermediate education	6	42.9	2	40		
Higher education	3	21.4	1	20		
Household						
Single	5	35.7				
Single and children	5	35.7				
Partner	1	7.1				
Partner and children	3	21.4				
Occupation						
No paid job or volunteer work	6	42.9				
Paid job or volunteer work	8	57.1				

Peer workers (*n* = 5) were mostly women, with varying educational backgrounds and with an average age of 58 years (range: 50–63 years). They worked as consultants, social workers, and public servants to improve support for people living in persistent poverty. All practitioners (*n* = 5) were female, received professional training in social work or a related professional area, and were, on average, 40 years old (range: 29–51 years). Practitioners supported people living in persistent poverty as counsellors, consultants, and coaches, for instance, with legal issues, debt, and finances. One practitioner also had experienced persistent poverty at some point in her life.

### Themes Regarding Wishes, Barriers, and Support Needs

Initially, multiple people living in persistent poverty found it difficult to express their wishes. Some indicated they had never really thought about their wishes for the future, which peer workers and practitioners explained was a result of having no “cognitive space,” that is, no mental capacity and resources to engage with and process information, make decisions, and plan for the future. Others deliberately chose not to dream as a form of self-preservation, explaining that daring to have wishes would only cause disappointment when they could not be fulfilled. However, as the interviews progressed, all people living in persistent poverty managed to formulate at least one wish.

You don’t have the freedom to dream because—yes, that’s very scary. Then you dream about something you cannot make come true, something that is unattainable. That becomes very painful. You may do that for a while in your younger years, but you quickly learn to stop dreaming, because it’s just not achievable when you have little money and don’t count. (Peer worker, 02601)

**Figure 1.**
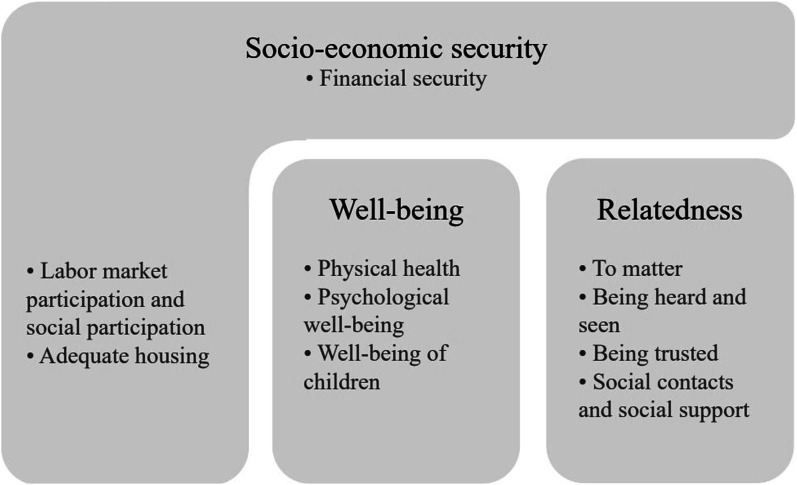
Themes and subthemes regarding the wishes of people living in persistent poverty.

From the wishes expressed by people living in persistent poverty, peer workers, and practitioners, three overarching themes emerged. These themes concern socio-economic security, well-being, and relatedness, which are visualized in Figure 1. Socio-economic security refers to wishes regarding individuals’ financial security, living conditions, and participation; well-being indicates wishes regarding physical and psychological well-being of oneself and of loved ones; and relatedness specifies wishes regarding individuals’ relationships with themselves and their environment, including network members, (governmental) organizations, and society at large. These wishes are discussed in relation to the barriers that obstruct them and the support they need for realizing these wishes.

#### Socio-Economic Security

##### Wishes

Almost all participants expressed that people living in persistent poverty wished for financial security. This means they wished to experience peace of mind regarding their finances and to have enough money to fulfill their basic needs, including paying their bills, buying food, using necessary healthcare, and supporting their children’s needs.

Still, all I want is just a job and sufficient income to live a normal life, without having to use the calculator ten times to see: “oh, what do I still have to pay?”, and “oh, what do I have left? Can I get a winter coat or shoes this month?” Just that you’re always preoccupied with money. Money, money, money, money. (Person living in persistent poverty, 01401)

Financial security was considered one of the most important wishes of people living in persistent poverty, as it was mentioned to be a prerequisite for realizing their other wishes:

You always need to have income if you want to move forward. If you don’t have money, you won’t survive. That’s what it always comes down to. If I don’t have money, I can’t live a healthy life, I can’t buy expensive vegetables. If I don’t have money, I can’t use healthcare, because I can’t pay my health insurance. I can’t exercise, I can’t … I could list everything. I can’t go to my family and friends by car, because they live 170 kilometers away. I can’t afford gas. My partner—well, you don’t feel like having a relationship because your situation is already so terrible at home, so to speak. Children—yeah, they’re great, but I don’t have time for them because my head is full. So I take it all out on my children. […] If there is no income, you can’t do anything. (Person living in persistent poverty, 01501)

In addition, participants often indicated they wanted to be (self-)employed, do volunteer work, or follow a study program or training course. These wishes for labor market participation and social participation were most often described as means to achieve financial security, but also served to fulfill wishes for psychological well-being and relatedness, including having structure in their daily lives, feeling useful, having a purpose, stimulating personal development, being a role model for their children, feeling of value, and having social interactions. In addition, participants expressed they wished to participate in hobbies, sports, and social activities.

Interviewer: Why is having a routine [...] so important to you?

Participant: [...] I think I’ve have been working since I was 15. And I’ve never been without work for this long, and I also notice that, yeah, then I stay in bed until 11 am or 4 pm, because there’s nothing else to do. And you have no reason to get up and you feel bad about everything. You worry. So, in order to have motivation again, to have work and to get up on time and get into that routine. To do it for something again. (Person living in persistent poverty, 01502)

Furthermore, some participants mentioned they wanted to improve the livability of their homes (e.g., adding necessary furniture and painting walls), to be able to cohabitate with their partner, and to move to another house, for instance, to better fit their family or regain a sense of safety.

##### Barriers

First, participants explained that people living in persistent poverty are hindered from realizing financial security because their income (i.e., minimum wage, welfare benefits, and weekly allowances) is simply too low to cover the basic costs of living. In addition, while some participants mentioned their strong budgeting skills and creative financial solutions, others discussed the trouble they had in handling the little amount of money they had. They, for instance, made financial decisions that were beneficial in the moment (e.g., to feel good or pay off a debt while having insufficient funds) but not in the long run, or did not do their financial administration out of fear of failing or facing their financial situation.

Another barrier for realizing financial security pertains to the type of available financial support as well as the limited offer of access to and use of financial support. Some commented that certain measures, like the possibility to request a laptop when needed, were merely seen as poverty management and were insufficient to structurally improve individuals’ financial situation. Others shared they “fell through the cracks,” as those who live on or just above the official poverty line did not qualify for any form of financial support, or because initiatives were available for children only. Moreover, participants often did not use available financial support. Some were not aware of all the measures they were entitled to; others were discouraged by the amount and complexity of administrative work it took to apply. In addition, long processing times before support is granted, geographical remoteness of support organizations, participants’ wish to be self-reliant, to maintain a sense of pride and autonomy, feelings of shame about their financial situation, and feelings of distrust and disappointment toward local authorities and support organizations also hindered them in using available support.

Right at the beginning when I was on welfare, I once requested exemption from municipal taxes and things like that. Let me tell you, that is a lot of work. Paperwork. I understand that they don’t make it too easy, but it takes a really long time and it’s a lot of work. And in the end, it’s rejected. So, on the one hand, I’m fortunate enough to be familiar with the Internet and I do read up on things [on the available support options]. I can imagine that people who are less educated, may not know about many of the rules. And on the other hand, the rules that do exist, are so complicated that at some point, you just think: never mind. (Person living in persistent poverty, 01101)

Another barrier in achieving financial security is participants’ limited access to the labor market. One practitioner described welfare benefits as an uncomfortable yet predictable zone. Some welfare recipients have reached a state of acceptance and resignation and have given up their wish to leave this zone. Some still wish for employment but are unable to motivate themselves to search for jobs or are unsure where and how to start. One participant, discussing why she needed help with starting her own business, explained:

Because in your head you have so many plans of action, right, so many things that need to be dealt with, that you don’t even know where to begin. (Person living in persistent poverty, 01202)

For others, finding a job is hindered by a sense of fear; fear of doing things they are not used to doing, fear of failing, fear of complex rules and procedures they do not understand, and fear of financial insecurity. Peer workers and practitioners explained that having a job implies that participants lose sight of exactly how much monthly income they will receive and that some will even experience a decline in income (i.e., the poverty trap). Participants also mentioned that rejections from potential employers, discrimination (ageism in particular), physical and psychological health problems (e.g., lack of energy and chronic pain), and the task of taking care of children and/or ill relatives hindered them in finding a job.

Afraid of what is to come. You see, if you’ve been on welfare for four years, it’s also very predictable what you’ll get every month. The moment you start working, yes, what can you expect? You are less entitled to benefits, you are expected to show up at work. So it’s also very scary to take the next step. (Practitioner, 02101)

Other wishes for social participation (i.e., education, volunteering, sports, hobbies, and social activities) and for adequate housing were mainly hindered by participants’ lack of financial means. Participants also mentioned that certain welfare laws—especially for cost sharing, which entails that households receive lower allowances the more cost sharers it counts—obstructed their housing wishes. One participant, for instance, was forced to live separately from her partner, as lower allowances made cohabitating financially unfeasible.

##### Support Needs

First, participants stressed the urgency of structural solutions to ensure financial security. Most importantly, they suggested raising individuals’ income, for instance, by increasing the minimum wage and welfare benefits, increasing the weekly allowance of individuals in debt settlements, or introducing a universal basic income. A practitioner also proposed that fixed expenses should be subtracted from social welfare benefits to ensure clarity and financial security.

In addition, participants pointed out the importance of preventative support in realizing financial security. This, for instance, implies that municipalities should reach out to individuals with arrears and that educational programs should teach minors about healthy financial behavior, their rights, and obligations. Furthermore, they argued that support should be tailor-made—that is, adapted to the needs of each individual—to realize more durable solutions to financial insecurity. This may, for instance, include reimbursing a scooter so individuals are able to commute or providing more opportunities to pursue education and follow courses.

At the moment, I have a client [...] who’s currently in debt restructuring. She’s on sick leave now and needs physiotherapy to recover. But physiotherapy isn’t covered [by her basic health insurance]. If it were covered, she could return to work more quickly and generate more income to pay off her debts. But because it isn’t covered, it’s likely that she’ll be on sick leave long-term and so less [money] goes to creditors. You hear these types of stories every day. (Practitioner, 02201)

Participants also underlined the importance of improving access to existing support structures. Some advocated for national coordination of support—as opposed to the current municipal coordination—in order to create equality in and clarity about the support participants are entitled to. They also emphasized support should be easily accessible, visible, and understandable, for instance, by creating an overview of all existing forms of support and regulations and by ensuring access does not require any or only minimal administrative work. Furthermore, the coordination between support organizations should be improved. Participants, for instance, stated that practitioners should refer participants to relevant forms of (financial) support, that one practitioner must be appointed to coordinate all supporting practitioners when participants receive assistance from multiple organizations, and that participants should have a single continuous file that is shared between organizations so participants do not have to start over each time they receive support.

#### Well-Being

##### Wishes

Many participants expressed that people living in persistent poverty wished for an improvement in their physical health, for instance, to experience less pain, quit smoking, exercise more, and have better dental health. Participants also wished for a better psychological well-being, including more peace of mind, more structure and purpose in life, more opportunities for personal development, as well as more autonomy and mastery. Regarding the latter, participants, for instance, shared they wanted freedom to make their own choices, more mobility, and to be (financially) independent. Moreover, some wished for a better ability to control their behavior (e.g., regarding smoking) and expressed the need for people living in persistent poverty to regain a sense of hope and perspective, often referred to as a “light at the end of the tunnel.”

A lot of people don’t see a future, especially when they have been living in poverty for a long time. They just don’t see any positive future prospects anymore. [...] So it’s very important to figure out how we can create some future perspective for them. (Practitioner, 02201)

In addition, participants wished for their children’s well-being, explaining they wanted their children to grow up healthily and happily, without being hindered in their development or being excluded from activities because of their financial situation.

##### Barriers

First, well-being wishes are hindered by a lack of financial resources. This obstructs access to healthy food and to healthcare that is not included in basic health insurance (e.g., physiotherapy and dental care) and impedes individuals’ peace of mind, hope for the future, independence and autonomy, and the opportunities they can offer their children.

Many families, both single-parent and two-parent households, want to provide for their children and give them the best opportunities possible. However, when you have limited financial resources, you don’t have the choice to make the best decisions for your children. You can’t choose the best school, for example, because you may not be able to afford transportation costs. This lack of choice affects all areas of life. (Peer worker, 02601)

Furthermore, the interviews highlighted that the well-being wishes were interrelated: An unfulfilled well-being wish was often considered an important barrier to realize other well-being wishes. Participants, for instance, shared that chronic stress, caused by the accumulation of stressors (e.g., having too little money, health problems of oneself and loved ones, relational problems, and behavioral problems), induced unhealthy habits (e.g., stress eating and smoking), physical problems (e.g., pain), and/or psychological problems (e.g., “a heavy mental burden” and burn-out). Moreover, the majority of participants mentioned that chronic stress and trauma, feelings of hopelessness and being stuck, and fear and expectance of setbacks and disappointment consumed all of their energy. Participants were consequently unable to turn their ideas into action and do the things that would make them feel better, varying from taking a walk to getting much needed support.

Due to all the stress and persevering all these years. And thinking: “Oh, next year will be better. Maybe .... You’ll make progress.” [...] And eventually you get tired and don’t take any initiative anymore to do things, because you don’t believe in it anymore. (Person living in persistent poverty, 01401)

I’m tired and exhausted from everything that happened lately. Just tired. And I also don’t feel like doing anything anymore. It’s not going to be okay. (Person living in persistent poverty, 01302)

##### Support Needs

To realize the well-being wishes of people living in persistent poverty, participants called for structural solutions, emphasizing that people living in persistent poverty should receive more income and that healthy food, physical and psychological healthcare, and healthcare insurance should be more affordable and accessible for everyone, regardless of income.

You have to ensure that people can live a decent life. That things are affordable. Because if you, let’s see, if you have to pay €200 per month for health insurance. Your income is say, €1100, and you have to pay say, €650, in rent, […] then you’ve already [almost] used up your entire income. You’ve [almost] used up your income on just two fixed expenses, and you haven’t even paid for your home insurance or other things yet. (Practitioner, 02401)

Moreover, they advocated for improving the quality of service provision. Multiple participants indicated that, in order to improve the well-being of people living in persistent poverty, support should be holistic and based on self-direction. Practitioners should gain insight into how participants are doing on each area of life, ask participants what they want and need, support participants toward realizing those wishes and needs, and let participants make decisions themselves. It was noted, moreover, that in most cases, support would have to start with creating peace of mind and cognitive space. Participants shared that getting an overview of their income and expenses, help with their financial administration, and debt restructuring procedures where a debt counsellor completely took over their financial affairs really helped them in this regard.

Self-direction. That they can make their own choices and that they always have a choice. Even if as professionals we sometimes seem to know what is best for our clients and we tend to present them with ready-made plans, and tell them that this is what they should do. But people just really want to have agency and want to have a choice. (Practitioner, 02201)

#### Relatedness

##### Wishes

People living in persistent poverty wished to feel that they matter, meaning they want to be valued just as much as anyone else, and to have more self-confidence. They also expressed the wish to be heard and seen, and to be trusted by others, specifically by local and national authorities and by care organizations.

I think people who live in poverty often […] feel like they’re a criminal because they receive welfare benefits. So, [people living in persistent poverty wish] that they matter, that they are not just a number, but a person with needs and wishes and dreams. (Practitioner, 02101)

Moreover, whereas some people living in poverty were content with their social network and social support, others articulated they wanted to interact with individuals outside their inner circle, to have more friends, or just have someone to talk to.

Interviewer: Are there any other things that come to mind that you want to change or attain?

Participant: Yes, I miss ... Yes, I’m just sitting at home, alone. And that’s it. Once a week I go visit my wife [who is in a nursing home]. Once a week I go grocery shopping. And the rest of the time I’m at home [...]. So yeah, that’s it. [...] What I miss, is just... Being able to share my thoughts and feelings. (Person living in persistent poverty, 01302)

##### Barriers

Participants indicated that feelings of mattering were affected by their upbringing, as some people living in persistent poverty had never learned to love themselves during their childhood. Insufficient financial resources also posed an important barrier for realizing relatedness wishes. Participants shared that having a low income in itself, as well as the resulting necessity to wear cheap or second-hand clothes and depend on (financial) support, made them feel “like beggars,” “discarded,” and as lesser human beings.

We went out for dinner. Not so posh, because all three of us were tight on money. And I thought: well, I don’t have to dress up as much. And then they wore glamorous dresses while I wore a denim skirt and second-hand boots. I felt so scrutinized. Especially because one of them [my sisters] looked at me with so much disdain. […] It made me feel so small that I thought: “Just give me money, so I can have a nice wardrobe again and look good.” (Person living in persistent poverty, 01101)

Participants’ stories also reveal that the (internalized) negative societal sentiment toward unemployment and poverty, and more direct experiences of stigmatization hindered their feelings of mattering. Multiple participants, for instance, experienced a sense of failing regarding expectations set by themselves and by society (e.g., “Why can’t I find a job”?), in comparison to others (e.g., other parents are able to pay for their child’s birthday party) and in comparison to their past selves (e.g., “I was good and now I am nothing”), inducing feelings of inferiority and shame. A practitioner summarized these psychological consequences of living in poverty as follows:

Look, poverty is not just a lack of money, but it also affects you internally. You experience social exclusion [and] stigmatization from others, and you also start treating yourself that way. So you grow up [in poverty] feeling like you’re worthless. And if you’re on welfare for a long time, you’re in a situation where you don’t feel like you have any right to exist, and there can be a lot of shame involved. (Practitioner, 02102)

Feelings of mattering were also hindered by the offer and provision of support. Specifically by the limited availability of support options for people living in persistent poverty and by the distrust ingrained in financial support. Participants, for instance, referred to the authoritarian and threatening tone of letters from local authorities; the requirement that social assistance recipients should justify the what and why for every bank statement; and the bulletproof glass in some meeting rooms, separating practitioners from (potentially aggressive) clients.

According to some peer workers, the barriers mentioned above are the result of a gap between the worlds of those living in poverty and those not living in poverty. They explained that society, policy makers, and practitioners have a limited understanding of the lived experiences of poverty and that people living in persistent poverty have limited knowledge to navigate the world of policy makers, fueling stigma and distrust.

Professionals just don’t know what it’s like to live in persistent poverty. You don’t experience it yourself. [...] And you may try to empathize, but you don’t know what it’s like to go through it every day. (Practitioner, 02201)

Lastly, participants’ wish for more social connectedness is mostly hindered by their lack of financial means. Participants described that people living in persistent poverty tend to have insufficient financial means to attend and travel to social activities (e.g., birthdays and family activities), making it harder for them to have and maintain social contacts with family, friends, and acquaintances.

##### Support Needs

To realize these relatedness wishes, participants highlighted several support needs regarding the approach of people living in persistent poverty by institutions, practitioners, local authorities, and society at large. First, they emphasized the importance of establishing institutional trust, for instance, by creating more lenient laws about receiving gifts and earning additional income. For this to be realized, participants argued the gap between the poor and non-poor should be closed. They suggested that knowledge about the impact of (chronic) stress and poverty on individuals’ lives should be more broadly disseminated to society—citizens, institutions, practitioners, and policy makers included—to facilitate an understanding of the lived experiences of poverty and reduce poverty stigma. Participants also suggested using peer workers in this regard, who were considered well-equipped to share their own story of poverty as well as collective poverty experiences and to voice the needs of people living in persistent poverty in policy making processes.

I want to advocate for more experts by experience at policy tables. Use that knowledge and expertise that are present there. How does a measure work out? What can be improved? [...] In an advisory board ... There are people who absolutely have no experience with poverty. They ask me: “what are you talking about?,” and then they’re baffled. And later, at the coffee machine, they say: “could you explain that again?”. You know. Yes, and that’s ... Yes, that’s all well and good, but these are the people who ... If you don’t have experience in the field, how can you give advice about it? (Person living in persistent poverty, 01202)

Second, they pointed out the importance of establishing a trust-based working alliance between practitioners and clients. This was considered crucial for realizing participants’ wish of being trusted in particular, as well as for participants to accept help. Establishing trust requires practitioners to take time for their clients, be patient and transparent, keep agreements, be easy to contact, and share some of their personal struggles. It also implies that practitioners need to give clients a sense of being heard and seen, by letting them share their story and their struggles, listening with interest and understanding, recognizing their hardship, and treating them as an equal, without prejudice. Contacts at local authorities, too, should adopt such an open and unprejudiced attitude toward people living in persistent poverty. In addition, participants vouched for using peer workers in service provision for people living in persistent poverty, too, as they were thought to be easy to trust and confide in, served as a beacon of hope, and showed them they were not alone.

## Discussion

This study is the first to examine the wishes, perceived barriers, and support needs of people living in persistent poverty in relation to one another. We drew on the perspectives of people living in persistent poverty, peer workers, and practitioners to gain a better understanding of what appropriate support for improving the quality of life of people living in persistent poverty entails.

We found that the wishes of people living in persistent poverty centered around improving socio-economic security (i.e., financial security, labor market participation and social participation, and adequate housing), improving the well-being of oneself and of loved ones (i.e., physical health, psychological well-being, and well-being of children), and improving relatedness (i.e., to matter, being heard and seen, being trusted, social contacts and social support). These wishes reflect multiple of the basic (psychological) needs that are considered vital for human well-being and growth (e.g., Nussbaum, 2000). This highlights that even in a prosperous state as the Netherlands, these universal basic needs are unattainable for people living in persistent poverty. It also emphasizes that poverty is not just a lack of financial means, but equates a lack of opportunity and freedom to pursue one’s wishes and improve one’s quality of life (also see Sen, 1999).

Our findings show that people living in persistent poverty were unable to realize their wishes as a result of four types of barriers: socio-economic barriers, support structures, an intergroup reality gap, and psychological barriers. The socio-economic barrier refers to individuals’ lack of money and is one of the most important barriers. Most wishes require money to be realized; the stress from not having enough money negatively affects individuals’ well-being and drains the energy needed for pursuing wishes; a lack of money affects one’s view of self, both directly and indirectly via one’s appearance (e.g., second-hand clothes); and it hinders individuals from maintaining and building social relations. This concurs with the importance Maslow (1943) ascribed to (financial) safety and security for being able to realize other wishes (but it does not demonstrate that the wishes of people living in persistent poverty follow a definite hierarchical order). It is also in line with previous empirical studies on the deteriorating impact of financial hardship on well-being (e.g., Frankham et al., 2020; Marttila et al., 2013; Richardson et al., 2013). Consequently, people living in persistent poverty, peer workers, and professionals advocated for structural solutions to reduce financial insecurity, including a focus on preventative support, a price reduction for healthy food, and a nation-wide raise of the minimum wage and welfare benefits. Previous research in both low-, middle-, and high-income countries accordingly demonstrates that experiments with basic income and cash transfers are associated with an increase in subjective well-being and mental health (Gibson et al., 2020; McGuire et al., 2022).

Barriers regarding the existing support structures include financial support aimed at poverty management rather than poverty eradication; financial support grounded in distrust toward the receiver; welfare laws that serve as additional stressors; and unavailable and insufficiently accessible service provision and healthcare for people living in persistent poverty. These factors perpetuate the cycle of poverty and social exclusion. They contribute to the negative societal sentiment and exacerbate individuals’ socio-economic insecurity (e.g., persistent financial insecurity), well-being (e.g., more stress and lack of adequate physical healthcare), and relatedness (e.g., not feeling valued). Therefore, in line with the literature, it was first argued that the so-called “administrative burden” of support should be minimized (also see Moynihan et al., 2015). This includes national coordination of support, a simple and clear overview of the available services, and easy access to service provision.

Furthermore, it was argued that people living in persistent poverty would be helped by institutional trust. Public policies, legislation, and services, in other words, should depart from the premise that all these individuals wish to improve their situation, and should be tailored to individuals’ situation and wishes. Wishes are key motivators (Lai et al., 2021; Wolf & Jonker, 2020) and are associated with better well-being and performance (Deci & Ryan, 2000; Sen, 1999; Sheldon et al., 2004; Vansteenkiste et al., 2004). Recent findings of a social experiment in the municipality of Nijmegen, the Netherlands, demonstrate that the most vulnerable social assistance recipients in particular tend to benefit from a more trust-based social assistance in terms of their experienced health and participation (Betkó, 2023).

In addition, for practitioners, our findings highlight the importance of adopting a holistic approach, of offering self-direction to clients, and of establishing a high-quality working alliance between practitioners and clients for realizing the wishes of people living in persistent poverty. Practitioners should be empathic and open minded, listen to and respect the wishes of their clients, and establish a relationship of trust and equality (also see O’Donnell et al., 2016; Williamson et al., 2006). Consistently, strengths-based approaches, which accentuate self-direction, a focus on strengths and assets, and a trust-based relationship, have been related to various positive outcomes for individuals in vulnerable positions (Fukui et al., 2012; Krabbenborg et al., 2017).

The difficulties people living in persistent poverty experience from the existing support structures seem to be the result of a gap between the realities and experiences of people living in persistent poverty and those who have no personal experience of living in poverty, including many policymakers, practitioners, and the general public. To close this gap, individuals with lived experiences of poverty should be actively involved in policy development and in the daily practice of service provision. This concurs with the growing awareness of the importance of listening to the voices of lived experiences, as an integral part of evidence-based policy development (Smith-Merry, 2020). Also within the daily practice of service provision, it is increasingly recognized that peer workers can offer a kind of support that other practitioners cannot or do not provide, and have the ability to “close the gap” between the reality of clients and that of practitioners (Van der Kooij & Keuzenkamp, 2018).

Lastly, people living in persistent poverty were hindered by psychological barriers in realizing their wishes. Our results showed that the various stressors and setbacks in the lives of people living in persistent poverty drained their energy, hope, and motivation to have and pursue wishes. They were also reportedly hindered by trauma, feelings of inferiority, and failure, and by a strong sense of fear. These psychological processes and “emotional costs” of poverty have also been documented by previous studies on the psychology of poverty and the sociology of emotions (e.g., Haushofer & Fehr, 2014; Underlid, 2007). These findings, moreover, emphasize the importance of establishing a trust-based working alliance between people living in persistent poverty and practitioners, and providing support that is tailored to the emotional needs of recipients (e.g., trauma-informed and stress-sensitive support).

It is important to note that the identified barriers interact with and reinforce each other, aggravating the disadvantaged situation of people living in persistent poverty in which they cannot realize their wishes for basic needs. For instance, a lack of appropriate support structures that tackle the root causes of poverty (support barrier) contributes to persistent financial insecurity (socio-economic barrier), which leads to chronic stress, which, in turn, undermines psychological well-being and impairs cognitive functions necessary for effective decision-making and the pursuit of long-term goals (psychological barriers). This is consistent with theories on scarcity (Shah et al., 2012). Similarly, institutional policies grounded in distrust (support barrier, intergroup reality gap) and fear toward services (psychological barrier) reduce their willingness to engage with support services, thereby limiting the effectiveness of these services and exacerbating the financial situation of individuals (socio-economic barrier). Therefore, in order to fundamentally improve the quality of life of people living in persistent poverty, it is essential that the abovementioned recommendations are implemented together instead of separately.

### Strengths and Limitations

This study has several limitations that should be noted. First, we conducted a fixed number of interviews and analyzed the data subsequent to, rather than simultaneous with, data collection. As a result, relevant findings that emerged during data analysis were not systematically probed during data collection. Analysis of the interviews did, however, cease to yield new results, indicating we did reach saturation. Second, we defined persistent poverty using the expenses-based not-much-but-sufficient criterion (Goderis et al., 2019) rather than an income-based criterion (e.g., low-income threshold). While these definitions demarcate persistent poverty using a different monthly budget and duration, we believe our choice of definition has only minimally affected our sample, given that the recruiters’ professional judgement was used to assess whether individuals met our definition of persistent poverty and that the majority of participants had been living in poverty for much longer than 3 years. On the other hand, our strategy of purposeful sampling did likely result in a biased sample of people living in persistent poverty. Participants, for instance, all spoke Dutch, most were involved in some kind of support trajectory, and none lived in one of the four largest cities of the Netherlands. In addition, we only interviewed a few individuals with a migration background, whereas this group constitutes the majority of people living in persistent poverty in the Dutch population (Statistics Netherlands, 2023). We attempted to grasp the perspectives of a broader range of individuals by including interviews with practitioners and peer workers, but we may nevertheless have missed some perspectives and intercultural nuances (e.g., the wish to learn the Dutch language). Also, our sampling strategy could have led to a biased sample of practitioners. Gatekeepers may have selected practitioners known for their empathetic engagement with clients, or such practitioners may have been more willing to participate. This may also clarify the discrepancy between the reported reality gap between clients and practitioners on the one hand, and the overlap in wishes, barriers, and support needs discerned by clients and practitioners on the other.

Despite these limitations, this study provides a unique insight into the wishes of people living in persistent poverty and what they, peer workers, and practitioners think is needed to overcome perceived barriers and realize these wishes. As a result, this study provides a wide range of clues to local and national policy makers and practitioners for optimizing appropriate and effective support to improve the quality of life of people living in persistent poverty. Another strength of this study lies in our close collaboration with peer workers, who were involved in the development of information letters, interview guides, and data collection. The reason for this was to start from the views of people living in poverty as much as possible and to examine their reality, challenges, and their understanding of solutions as closely as possible.

### Conclusion

Our study showed that structural changes in policy and practice are needed for people living in persistent poverty to fulfill their wishes for socio-economic security, well-being, and relatedness. These structural changes should provide people living in persistent poverty more financial security and better access to appropriate support and healthcare, and should close the reality gap between those who live in persistent poverty on the one hand, and policy makers, practitioners, and the general public on the other. In addition, the quality of the working alliance between practitioners and people living in persistent poverty should have a central role in service provision (e.g., based on trust, equality, empathy, and open-mindedness). These changes should be implemented together in order to effectively support people living in persistent poverty in improving their quality of life.

## References

[bibr1-10497323241309803] Betkó J. (2023). Effects of welfare policies based on autonomy and unconditionality: A social experiment with social assistance recipients [Doctoral dissertation]. Radboud University Nijmegen. Radboud Repository. https://repository.ubn.ru.nl/bitstream/handle/2066/290385/290385.pdf?sequence=1

[bibr2-10497323241309803] Braun V. Clarke V. (2006). Using thematic analysis in psychology. Qualitative Research in Psychology, 3(2), 77–101. 10.1191/1478088706qp063oa

[bibr3-10497323241309803] Canvin K. Jones C. Marttila A. Burström B. Whitehead M. (2007). Can I risk using public services? Perceived consequences of seeking help and health care among households living in poverty: Qualitative study. Journal of Epidemiology & Community Health, 61(11), 984–989. 10.1136/jech.2006.058404 17933957 PMC2465616

[bibr4-10497323241309803] Crouch E. Jones J. Strompolis M. Merrick M. (2020). Examining the association between ACEs, childhood poverty and neglect, and physical and mental health: Data from two state samples. Children and Youth Services Review, 116, Article 105155. 10.1016/j.childyouth.2020.105155

[bibr5-10497323241309803] Deci E. L. Ryan R. M. (2000). The ‘what’ and ‘why’ of goal pursuits: Human needs and the self-determination of behavior. Psychological Inquiry, 11(4), 227–268. 10.1207/S15327965PLI1104_01

[bibr6-10497323241309803] Doyal L. Gough I. (1984). A theory of human needs. Critical Social Policy, 4(10), 6–38. 10.1177/026101838400401002

[bibr7-10497323241309803] Eurostat . (2024). At risk of poverty rate [Data set]. 10.2908/ILC_LI02

[bibr8-10497323241309803] Evans G. W. Kim P. (2012). Childhood poverty, chronic stress, self-regulation, and coping. Child Development Perspectives, 7(1), 43–48. 10.1111/cdep.12013

[bibr9-10497323241309803] Frankham C. Richardson T. Maguire N. (2020). Do locus of control, self-esteem, hope and shame mediate the relationship between financial hardship and mental health? Community Mental Health Journal, 56(3), 404–415. 10.1007/s10597-019-00467-9 31552540 PMC7056732

[bibr10-10497323241309803] Fukui S. Goscha R. Rapp C. Mabry A. Liddy P. Marty D. (2012). Strengths model case management fidelity scores and client outcomes. Psychiatric Services, 63(7), 708–710. 10.1176/appi.ps.201100373 22752035

[bibr11-10497323241309803] Gibson M. Hearty W. Craig P. (2020). The public health effects of interventions similar to basic income: A scoping review. The Lancet Public Health, 5(3), E165–E176. 10.1016/S2468-2667(20)30005-0 32113520 PMC7208547

[bibr12-10497323241309803] Goderis B. Van Hulst B. Hoff S. (2019). Waar ligt de armoedegrens? [What is the poverty threshold?] SCP. https://digitaal.scp.nl/armoedeinkaart2019/waar-ligt-de-armoedegrens/

[bibr14-10497323241309803] Gupta A. Blumhardt H. (2016). Giving poverty a voice: Families’ experiences of social work practice in a risk-averse child protection system. Families, Relationships and Societies, 5(1), 163–172. 10.1332/204674316X14540714620166

[bibr15-10497323241309803] Haushofer J. Fehr E. (2014). On the psychology of poverty. Science, 344(6186), 862–867. 10.1126/science.1232491 24855262

[bibr17-10497323241309803] Kahn J. R. Pearlin L. I. (2006). Financial strain over the life course and health among older adults. Journal of Health and Social Behavior, 47(1), 17–31. 10.1177/002214650604700102 16583773

[bibr18-10497323241309803] Krabbenborg M. Boersma S. Van der Veld W. Van Hulst B. Vollebergh W. Wolf J. (2017). A cluster randomized controlled trial testing the effectiveness of Houvast: A strengths-based intervention for homeless young adults. Research on Social Work Practice, 27(6), 639–652. 10.1177/1049731515622263

[bibr19-10497323241309803] Lai D. W. L. Chan K. C. Daoust G. D. Xie X. J. (2021). Hopes and wishes of clients with mentally illness in Hong Kong. Community Mental Health Journal, 57(8), 1556–1565. 10.1007/s10597-021-00779-9 33507470 PMC7841978

[bibr20-10497323241309803] Loignon C. Hudon C. Goulet E. Boyer S. De Laat M. Fournier N. Grabovschi C. Bush P. (2015). Perceived barriers to healthcare for persons living in poverty in Quebec, Canada: The EQUIhealThY project. International Journal for Equity in Health, 14(4), Article 4. 10.1186/s12939-015-0135-5 25596816 PMC4300157

[bibr21-10497323241309803] Mani A. Mullainathan S. Shafir E. Zhao J. (2013). Poverty impedes cognitive function. Science, 341(6149), 976–980. 10.1126/science.1238041 23990553

[bibr22-10497323241309803] Marttila A. Johansson E. Whitehead M. Burström B. (2013). Keep going in adversity – Using a resilience perspective to understand the narratives of long-term social assistance recipients in Sweden. International Journal for Equity in Health, 12(8), Article 8. 10.1186/1475-9276-12-8 23339587 PMC3560245

[bibr23-10497323241309803] Marttila A. Whitehead M. Canvin K. Burström B. (2010). Controlled and dependent: Experiences of living on social assistance in Sweden. International Journal of Social Welfare, 19(2), 142–151. 10.1111/j.1468-2397.2009.00638.x

[bibr24-10497323241309803] Maslow A. H. (1943). A theory of human motivation. Psychological Review, 50(4), 370–396. 10.1037/h0054346

[bibr25-10497323241309803] McGuire J. Kaiser C. Bach-Mortensen A. M. (2022). A systematic review and meta-analysis of the impact of cash transfers on subjective well-being and mental health in low- and middle-income countries. Nature Human Behaviour, 6(3), 359–370. 10.1038/s41562-021-01252-z 35058643

[bibr26-10497323241309803] Mood C. Jonsson J. O. (2016). The social consequences of poverty: An empirical test on longitudinal data. Social Indicators Research, 127, 633–652. 10.1007/s11205-015-0983-9 27239091 PMC4863915

[bibr27-10497323241309803] Morse J. M. (2010). How different is qualitative health research from qualitative research? Do we have a subdiscipline? Qualitative Health Research, 20(11), 1459–1464. 10.1177/1049732310379116 20693515

[bibr28-10497323241309803] Moynihan D. Herd P. Harvey H. (2015). Administrative burden: Learning, psychological, and compliance costs in citizen-state interactions. Journal of Public Administration Research and Theory, 25(1), 43–69. 10.1093/jopart/muu009

[bibr29-10497323241309803] Nussbaum M. (2000). Women and human development: A study in human capabilities. Cambridge University Press.

[bibr30-10497323241309803] O’Donnell P. Tierney E. O’Carroll A. Nurse D. MacFarlane A. (2016). Exploring levers and barriers to accessing primary care for marginalised groups and identifying their priorities for primary care provision: A participatory learning and action research study. International Journal for Equity in Health, 15(1), Article 197. 10.1186/s12939-016-0487-5 27912783 PMC5135741

[bibr31-10497323241309803] Price J. H. Khubchandani J. Webb F. J. (2018). Poverty and health disparities: What can public health professionals do? Health Promotion Practice, 19(2), 170–174. 10.1177/1524839918755143 29363333

[bibr32-10497323241309803] Price R. H. Choi J. N. Vinokur A. D. (2002). Links in the chain of adversity following job loss: How financial strain and loss of personal control lead to depression, impaired functioning, and poor health. Journal of Occupational Health Psychology, 7(4), 302–312. 10.1037//1076-8998.7.4.302 12396064

[bibr33-10497323241309803] Richardson T. Elliott P. Roberts R. (2013). The relationship between personal unsecured debt and mental and physical health: A systematic review and meta-analysis. Clinical Psychology Review, 33(8), 1148–1162. 10.1016/j.cpr.2013.08.009 24121465

[bibr35-10497323241309803] Sen A. (1999). Development as freedom. Anchor Books.

[bibr36-10497323241309803] Shah A. K. Mullainathan S. Shafir E. (2012). Some consequences of having too little. Science, 338(6107), 682–685. 10.1126/science.1222426 23118192

[bibr37-10497323241309803] Sheldon K. M. Ryan R. M. Deci E. L. Kasser T. (2004). The independent effects of goal contents and motives on well-being: It’s both what you pursue and why you pursue it. Personality & Social Psychology Bulletin, 30(4), 475–486. 10.1177/0146167203261883 15070476

[bibr38-10497323241309803] Smith-Merry J. (2020). Evidence-based policy, knowledge from experience and validity. Evidence & Policy, 16(2), 305–316. 10.1332/174426419X15700265131524

[bibr39-10497323241309803] Statistics Netherlands . (2023). Laag en langdurig laag inkomen van huishoudens; huishoudenskenmerken [Households with low and persistently low income; household characteristics] [Data set]. https://opendata.cbs.nl/statline/#/CBS/nl/dataset/83841NED/table?ts=1715880319953

[bibr40-10497323241309803] Taylor M. J. Barusch A. S. (2004). Personal, family, and multiple barriers of long-term welfare recipients. Social Work, 49(2), 175–183. 10.1093/sw/49.2.175 15124958

[bibr41-10497323241309803] Thomson R. M. Igelström E. Purba A. K. Shimonovich M. Thomson H. McCartney G. Reeves A. Leyland A. Pearce A. Katikireddi S. V. (2022). How do income changes impact on mental health and wellbeing for working-age adults? A systematic review and meta-analysis. The Lancet Public Health, 7(6), e515–e528. 10.1016/S2468-2667(22)00058-5 35660213 PMC7614874

[bibr42-10497323241309803] Underlid K. (2007). Poverty and experiences of insecurity. A qualitative interview study of 25 long-standing recipients of social security. International Journal of Social Welfare, 16(1), 65–74. 10.1111/j.1468-2397.2006.00423.x 15842418

[bibr43-10497323241309803] Van der Kooij A. Keuzenkamp S. (2018). Ervaringsdeskundigen in het sociaal domein: Wie zijn dat en wat doen ze? [Peer workers in the social domain: Who are they and what do they do?] Movisie. https://www.movisie.nl/sites/movisie.nl/files/2019-11/Startnotitie-Ervaringsdeskundigen-in-het-sociaal-domein.pdf

[bibr45-10497323241309803] Vansteenkiste M. Simons J. Lens W. Sheldon K. M. Deci E. L. (2004). Motivating learning, performance, and persistence: The synergistic effects of intrinsic goal contents and autonomy-supportive contexts. Journal of Personality and Social Psychology, 87(2), 246–260. 10.1037/0022-3514.87.2.246 15301630

[bibr46-10497323241309803] Washington State, Department of Children, Youth, and Families . (2018). Mobility Mentoring® outcomes report 2017-2018. https://www.dcyf.wa.gov/services/early-learning-providers/eceap/reports

[bibr47-10497323241309803] Watson J. Crawley J. Kane D. (2016). Social exclusion, health and hidden homelessness. Public Health, 139, 96–102. 10.1016/j.puhe.2016.05.017 27340041

[bibr48-10497323241309803] Williamson D. L. Stewart M. J. Hayward K. Letourneau N. Makwarimba E. Masuda J. Raine K. Reutter L. Rootman I. Wilson D. (2006). Low-income Canadians’ experiences with health-related services: Implications for health care reform. Health Policy, 76(1), 106–121. 10.1016/j.healthpol.2005.05.005 15978694

[bibr49-10497323241309803] Wolf J. R. L. M. Jonker I. E. (2020). Pathways to empowerment: The social quality approach as a foundation for person-centered interventions. International Journal of Social Quality, 10(1), 29–56. 10.3167/IJSQ.2020.100103

